# Attachment style contributes to the outcome of a multimodal lifestyle intervention

**DOI:** 10.1186/1751-0759-6-3

**Published:** 2012-02-02

**Authors:** Sybille Kiesewetter, Andrea Köpsel, Knut Mai, Andrea Stroux, Thomas Bobbert, Joachim Spranger, Werner Köpp, Hans-Christian Deter, Bettina Kallenbach-Dermutz

**Affiliations:** 1 Department of Psychosomatics and Psychotherapy, Charité - Universitätsmedizin Berlin, Campus Benjamin Franklin, Hindenburgdamm 30, 12200 Berlin, Germany; 2 Department of Endocrinology, Diabetes and Nutrition, Charité - Universitätsmedizin Berlin, Campus Benjamin Franklin, Hindenburgdamm 30, 12200 Berlin, Germany; 3 Department of Epidemiology and Statistics, Charité - Universitätsmedizin Berlin, Campus Benjamin Franklin, Hindenburgdamm 30, 12200 Berlin, Germany

**Keywords:** attachment style, obesity, patient-therapist relationship, weight reduction

## Abstract

**Background & Aims:**

The long-term success of life-style interventions in the treatment of obesity is limited. Although psychological factors have been suggested to modify therapeutic effects, specifically the implications of attachment styles and the patient-therapist relationship have not been examined in detail yet.

**Methods:**

This study included 44 obese patients who participated in a one-year multimodal weight-reduction program. Attachment style was analyzed by the Adult Attachment Prototype Rating (AAPR) inventory and its relation to a one-year weight reduction program was studied. The patient-therapist-relationship was assessed using the Helping Alliance Questionnaire.

**Results:**

Attachment style was secure in 68% of participants and insecure (preoccupied and dismissing) in 32%. Interestingly a significantly higher weight-reduction was found in securely (SAI) compared to insecurely attached individuals (UAI; p < 0.05). This estimation correlated positively also to the quality of helping alliance (p = 0.004).

**Conclusions:**

The frequency of insecure attachment in obese individuals was comparable to that of the normal population. Our data suggest a greater weight-reduction for SAI than for UAI, and the patient-therapist relationship was rated more positively. The conclusion can be drawn that a patient's attachment style plays a role in an interdisciplinary treatment program for obesity and has an influence on the effort to lose weight.

## Introduction

The incidence of obesity is increasing worldwide. Data from the German microcensus conducted in 2005 revealed that about 58% of adult men and 42% of adult women are overweight (Body Mass Index ≥ 25 kg/m^2^) [1].

This is of special importance given that obesity is one of the major risk factors for diabetes, cardiovascular disorders and cancer [2-4].

Although large intervention trials were able to demonstrate that a multimodal life-style intervention can prevent type 2 diabetes mellitus in individuals at risk, the long-term effect of those interventions on body weight is limited [5,6]. The poor long-term success of weight reduction may be based on the fact that obesity is a complex disorder. Genetic and environmental parameters affect energy homeostasis [7,8]. Additionally, it is well known that psychological parameters have a substantial effect on the body weight and well being of the individuals [9,10]. Concerning the therapy of obese subjects, the relationship to the therapist may play an important role [11]. These relationships depend especially on earlier experiences and the attachment style of the patient. Therefore the benefit from a weight loss program may be related to the patient's attachment style and the ability of the patient to engage in group interaction [12].

Attachment theory assumes that humans and most mammals have a biologically predisposed attachment system [13]. This system is responsible for the strong emotional mother-child (primary caregiver-child) relationship [14]. It is activated as soon as an outer or inner danger arises that cannot be overcome by the child himself and thus has a survival-ensuring function. The so-called "attachment behavior" is triggered. The small child turns to a familiar person, e.g. his mother or father, to whom he develops a very specific attachment. His feelings, expectations and behavioral strategies are integrated into this attachment relationship, which develops during the first year of life. In its basic structure it is stable over time [15] and forms an emotional basis throughout life, although change in different directions through emotional experiences in new relationships or through psychotherapy is possible. Attachment styles are currently differentiated as secure, preoccupied, dismissing, fearful-avoidant, and unresolved [16]. This could have consequences especially in obese patients. A cofactor in some individuals with obesity is assumed to be immature affect regulation, which has its roots in the interaction with the primary caregivers [10] and is related to early attachment experiences. Bruch [9,17] stresses the motivational elements in the development of obesity and assumes that obese individuals are not able to distinguish feelings of hunger from other physical needs or from emotional tension. Therefore, we assumed that obese patients, like patients with other eating disorders [18,19] may have a more insecure attachment style compared to clinically normal subjects.

Compliance and adherence is usually poor in obese individuals [20,21], which may also be attributable to the attachment style of individuals as an important reason: Attachment strategies play an important role in the development of a therapeutic relationship [22]. Despite this potential impact on the therapeutic success of interventions, the significance of attachment styles for weight reduction therapy in obese individuals has not yet been investigated. We hypothesized that insecurely attached obese patients may lose less weight than securely attached patients (H1). Additionally the patient-therapist relationship may be assessed more positively by obese individuals with secure attachment than by those with insecure attachment (a) and is also assessed more positively by the therapist for the former than for the latter individuals (b).

## Method

### Subjects

44 volunteers (40 women and 4 men) participated in a weight reduction program for 12 months. Mean age was 52.3 ± 10.5 years. Mean starting weight was 108.2 ± 23.6 kg and a BMI of 38.3 ± 7.4 kg/m^2 ^respectively. All participants were screened for serious health problems and the intake of medication and were excluded if vascular diseases, hepatic or renal diseases or severe psychological co-morbidities were found. Further characteristics of the participants are provided in table 1.

**Table 1:** Further characteristics of the participants.

	total N = 44 (100%)		Insecure attachment style total N = 14 (32%)		Secure attachment style total N = 30 (68%)		
	**Mean**	**SD**	**Mean**	**SD**	**Mean**	**SD**	**p**

Age (years)	52.3	10.5	51.7	8.6	52.6	11.4	n.s.

BMI baseline (kg/m2)	38.3	7.4	40.6	9.1	37.2	6.4	n.s.

Weight reduction in% (BMI points)	6.3	6.2	3.5	8.0	7.6	4.7	0.047

Weight in kg baseline	108.2	23.6	116.0	22.3	104.5	23.7	0.034

Weight reduction in kg after 1 year	5.7	7.5	2.0	10.2	7.5	5.1	0.029

HAQ patient 3rd session	4.3	0.6	3.9	0.7	4.5	0.5	0.004

HAQ therapist 3rd session	3.6	0.6	3.4	0.5	3.8	0.6	0.019

Age = Age at the beginning of the program; BMI baseline = Body mass Index (BMI) at the beginning of the program; HAQ patient 3rd session = Helping Alliance total score of the patients after the 3^rd ^session; HAQ therapist 3rd session = Helping Alliance total score of the therapist after the 3^rd ^session

The experimental protocol was approved by the Institutional Review Board, and all subjects gave written informed consent. We certify that all applicable institutional and governmental regulations concerning the ethical use of human volunteers were followed during this research.

### Dietary and psychosocial intervention

All volunteers documented their eating behaviour for 3 days before intervention. Based on eating protocols, individual counselling was provided, with the recommendation of a daily calorie intake of 400-600 kcal less than the total energy expenditure. The diet was composed according to the guidelines of the German Association of Nutrition with the following distribution of macronutrients: carbohydrates 50%, fat 30% and protein 20% of the daily energy intake. Meetings for all volunteers took place over the first 6 months once a week in groups consisting of 10-12 participants with participation costs amounting to 450 €. In the first 90 minutes at the first 10 sessions, nutrition-consultants accomplished group workshops with practical cooking exercises. One workshop was provided by a physician with further hints and advise. At all dates, moderate exercise with gymnastics or aqua-fitness was performed during the last 60 minutes. The patients participated on average in 12 60- to 90-minute sessions of interactional group therapy [23]. This type of psychodynamic group therapy requires active treatment by the therapist. He or she uses supportive interventions such as giving advice, and often mirrors the acting-out of group members. Transference onto the therapist in this setting is not an explicit goal. Consequently, interactional group therapy is more adequate for these obese patients attending a weight reduction program and who mostly do not have a primarily psychotherapeutic objective.

In months 7 to 12, the participants had consultations with the nutritionists every 6-8 weeks. Anthropometry was determined in all participants before the dietary intervention and at the end of the program, after 12 months. All measurements were performed as previously described [24].

### Psychosocial parameters

Attachment style was determined in all participants before the start of the weight reduction by conducting semi-standardized one- to two-hour attachment interviews with the patients using the German version of the Adult Attachment Prototype Rating (AAPR) [25] inventory. The interviewers had previously undergone intensive rater training. The AAPR has demonstrated its reliability and validity in a variety of studies [26]. It shows satisfactory psychometric features with respect to inter-rater reliability, internal consistency of scales, and discriminatory power of the individual items [27]. Analysis focused on the three organized attachment styles (secure, preoccupied and dismissing) found in the individuals of this study: Securely attached adults experienced in their childhood the primary caregivers as acting and reacting sensitively and promptly and were therefore able to develop a sense of basic trust as adults. Preoccupied attached adults experienced during their childhood that the mother sometimes reacted adequately but sometimes not. This led to insecurity towards the primary caregiver regarding their availability during an emotional emergency. As adults, they show a tendency to cling and feel insecure. Adults with a dismissing attachment style mostly experienced insensitive or uncaring parents. As adults, they try to avoid affection and help from others, and strongly strive for autonomy.

The patient-therapist relationship was determined for all participants and the therapist after the 3^rd ^group session using the German version of the Helping Alliance Questionnaire (HAQ) [28,29]. The 11-item questionnaire comprises 2 subscales: 1) help received from the therapist, i.e. whether he or she really provides the help needed; 2) the therapist-patient relationship. Patients rate the applicability of the statements with regard to the two subscales on a 6-point Likert scale ranging from 1 ("No, I think this does not apply at all.") to 6 ("Yes, I think this fully applies.") without a neutral middle point.

Depression was assessed using the Brief Symptom Inventory (BSI) [30].

### Statistical analysis

All data are presented as mean (M) and standard deviation (SD) or the absolute and relative frequencies for categorical data. Success in weight reduction within one year has been quantified by changes relative to baseline data. Due to the skewed distribution of numerical variables, differences between groups were analyzed using the Mann-Whitney U test, or Wilcoxon signed rank test for comparisons between different time points. The Chi square test was used for group comparisons involving categorical data. Fisher's exact test was used instead if more than 25% of the cells had an expected cell size of less than five. To test whether or not the attachment style has an effect on weight reduction, a one way ANOVA was used. Coefficients are considered significant if the respective p-values are less than α = 0.05.

## Results

The total group of 44 obese patients (BMI ≥ 30) comprised 4 men (9%) and 40 women (91%) between the ages of 20 and 69 (52.3 ± 10.5). At the beginning of the weight reduction program, 39% of the patients had grade I (BMI = 30.0 - 34.9), 34% grade II (BMI = 35.0 - 39.9), and 27% grade III obesity (BMI ≥ 40.0). BMI ranged from 30.2 to 69.0 kg/m^2^.

In total, participants had a 6.3% reduction of body weight (with a range from 20.9% reduction to 11.2% weight gain) at month 12 compared to baseline (p < 0, 001).

Within the group of 44 patients, 30 (68%) were securely and 14 (32%) insecurely attached (6 (14%) with a preoccupied and 8 (18%) with a dismissing attachment style). At baseline, secure and insecure patients did not differ with respect to sex, age, or BMI, but did differ in regard to weight in kg at baseline (0.034, Table 1). In a subsample of patients with depression values the insecure and secure attached patients differed with respect to the depression-scale of the BSI at baseline (secure attached (N = 25): 52.7 (SD: 10.6); insecure attached (N = 11): 62.5 (SD: 11.4), p = 0.024).

Most interestingly, the weight loss was more pronounced for securely than for insecurely attached patients (7.6% vs. 3.5%, Table 1). The ANOVA confirmed that weight reduction among individuals with a secure attachment style was significantly higher than among the insecurely attached (p = 0.047) individuals. Taking the BMI at baseline into account, the effect of attachment style on weight reduction still shows a statistical tendency (p < 0.1).

The patient-therapist relationship (HAQ) was assessed more positively by securely than by insecurely attached patients (p = 0.004), and the relationship components were also assessed more positively by the therapist in securely attached patients (p = 0.019, Table 1). There was no significant association between weight loss and the ratings of the patient-therapist relationship (HAQ: patients rating p = 0.694, therapists rating p = 0.466).

## Discussion

Psychological factors have been postulated to be involved in the success of weight reduction programs. We here demonstrated that the attachment style of participants had an effect on the success of a multi-factorial lifestyle intervention in terms of weight reduction.

That insecure attachment was not more common among the obese patients in this study than has been reported in non-clinical individuals (securely attached: 60%; preoccupied: 17% dismissing: 23% [31]) suggests that obesity did not affect the occurrence of different attachment styles. But, there could be a selection bias. In a study with anorectic and bulimic patients Goodwin & Fitzgibbon [32] showed that insecurely attached eating-disordered patients often decline an outpatient treatment offer, which would also explain the high percentage of securely attached patients in our random sample: Insecurely attached subjects do not enroll in such a one-year group program in the first place. Therefore, our study was not representative, since voluntary participation has certainly biased results.

The sex ratio of our random sample of 91% women and 9% men approximately coincides with that of treatment-seeking obese populations reported in literature (85% women and 15% men; [33]). This could be attributed to treatment motivation being determined by social rejection, which applies mainly to obesity in women. The largest age group in this study comprised 40 - 59-year-olds. These individuals may have a stronger desire to lose weight. Another reason could be that they are more able and willing to afford the participation costs.

Compliance to weight reduction programs is usually poor in obese individuals and therefore they often regain weight [20]. Thus improved compliance might contribute to the therapeutic effect in those individuals with secure attachment style. This assumption is supported by the finding that the patient-therapist relationship was assessed to be more positive in secure compared to insecure participants. Indeed, previous publications confirmed that securely attached adults are considerably more cooperative in a patient-therapist situation [22]. This corresponds to observations indicating that securely attached individuals have better access to their emotions and eating habits, can therefore better perceive, understand and name them, and thus also have better prerequisites for successful weight reduction [23]. Individuals with an insecure attachment style are more likely to have problems with these issues; in the case of emotional entanglement, for example, preoccupied attached individuals do not perceive eating as an attempt to compensate. Dismissively attached individuals, on the other hand, tend to have difficulties in experiencing emotions and thus compensate by eating; in the case of therapeutic failure, they tend to blame others - in this setting the therapist.

It may be assumed that insecurely attached patients have difficulties in coping with the disease and also with time-limited therapies [34]. Thus the limited number of group therapy sessions offered in our setting might have negatively affected especially preoccupied attached patients, while securely attached patients were more stable in this respect. In addition, different reactions of the therapists, which are well known to occur and may have been induced by the patients' attachment style [11], may also have affected differences in weight loss.

Some limitations of our study should be mentioned: For estimating the distribution of attachment styles in representative, non-therapy seeking obese individuals compared to normal weight individuals, our study results are not representative and further studies are required. This trial was a clinical outcome study, it was not randomized and there was no control group. Predominantly women were studied; thus, the results can not be transferred to male subjects. Due to the small number of cases, we did not analyze the subgroups of binge eating, night or emotional eaters, and the effect of additional psychiatric co-morbidities in this study. To estimate the relevance of attachment styles according gender, age, and different subgroups, future studies are desirable. But there is a strong support for the assumption that the attachment style and the helping alliance between patient and physician are important in the therapy of obesity and probably other diseases.

## Competing interests

None of the authors have any financial or personal relationships with other people or organisations that could inappropriately influence (bias) our work.

## Authors' contributions

SK: carried out the psychodiagnostic part of the study, participated at the group therapy and drafted the manuscript. AK carried out the psychodiagnostic specification of the attachment style. KM was responsible for the analysis of somatic data of the obese patients. AS performed the statistical analyses. TB selected the obese patients for the study and carried out the medical examination. WK was supervisor of the attachment style judgments and carried out the interrater reliability measurements. HCD participated in the design and coordination of the study and was the PI. BKD gave support for the therapeutic activity of the study, supervised the group therapist and was responsible for all therapeutic activity in the outpatient clinic for eating disorders. All authors read and approved the final manuscript.
